# Late Holocene climatic variability in Subarctic Canada: Insights from a high-resolution lake record from the central Northwest Territories

**DOI:** 10.1371/journal.pone.0199872

**Published:** 2018-06-28

**Authors:** April S. Dalton, R. Timothy Patterson, Helen M. Roe, Andrew L. Macumber, Graeme T. Swindles, Jennifer M. Galloway, Jesse C. Vermaire, Carley A. Crann, Hendrik Falck

**Affiliations:** 1 Ottawa-Carleton Geoscience Centre and Department of Earth Sciences, Carleton University, Ottawa, Canada; 2 Department of Earth Sciences, University of Toronto, Toronto, Canada; 3 School of Natural and Built Environment, Queen’s University of Belfast, Belfast, United Kingdom; 4 School of Geography, University of Leeds, Leeds, United Kingdom; 5 Geological Survey of Canada / Commission géologique du Canada, Calgary, Alberta, Canada; 6 Natural Resources Canada / Ressources naturelles Canada, Geological Survey of Canada / Commission géologique du Canada, Calgary, Canada; 7 Institute of Environmental Science and Department of Geography and Environmental Studies Carleton University, Ottawa, Canada; 8 A.E. Lalonde AMS Laboratory, University of Ottawa, Ottawa, Canada; 9 Northwest Territories Geological Survey, Yellowknife, Canada; Indiana University-Purdue University Indianapolis, UNITED STATES

## Abstract

We examined late Holocene (ca. 3300 yr BP to present-day) climate variability in the central Northwest Territories (Canadian Subarctic) using a diatom and sedimentological record from Danny’s Lake (63.48ºN, 112.54ºW), located 40 km southwest of the modern-day treeline. High-resolution sampling paired with a robust age model (25 radiocarbon dates) allowed for the examination of both lake hydroecological conditions (30-year intervals; diatoms) and sedimentological changes in the watershed (12-year intervals; grain size records) over the late Holocene. Time series analysis of key lake ecological indicators (diatom species *Aulacoseira alpigena*, *Pseudostaurosira brevistriata* and *Achnanthidium minutissimum)* and sedimentological parameters, reflective of catchment processes (coarse silt fraction), suggests significant intermittent variations in turbidity, pH and light penetration within the lake basin. In the diatom record, we observed discontinuous periodicities in the range of ca. 69, 88–100, 115–132, 141–188, 562, 750 and 900 years (>90% and >95% confidence intervals), whereas the coarse silt fraction was characterized by periodicities in the >901 and <61-year range (>95% confidence interval). Periodicities in the proxy data from the Danny’s Lake sediment core align with changes in total solar irradiance over the past ca. 3300 yr BP and we hypothesize a link to the Suess Cycle, Gleissberg Cycle and Pacific Decadal Oscillation via occasional inland propagation of shifting air masses over the Pacific Ocean. This research represents an important baseline study of the underlying causes of climate variability in the Canadian Subarctic and provides details on the long-term climate variability that has persisted in this region through the past three thousand years.

## Introduction

General Circulation Models predict that near-future climate warming in Arctic and Subarctic regions will be of a greater magnitude than projected for lower latitudes [1]. To improve the predictive power of these models, and to put climate warming projections into the context of natural variability in the northern climate system, it is important to obtain empirically derived paleoclimate reconstructions from the region. Paleolimnological records are valuable contributors to this effort as they archive long-term climate information via sedimentological, geochemical and biological proxies, permitting reconstruction of climatic records that extend well beyond that recorded in the instrumental record. Lakes in Subarctic Canada cover up to 25% of the land surface [2] and represent important Holocene records for insight into landscape development and climate sensitivity (e.g. [3, 4]). Despite numerous studies on these widespread lacustrine records, our understanding of the long- and short-term climate dynamics that have influenced the Arctic and Subarctic regions remains incomplete [5–7]. As such, there is a need for additional regionally representative climate studies to understand more thoroughly long-term variability.

Lacustrine paleo-records in Subarctic Canada began when glacial ice receded from this region ca. 12000 to 8000 years before present (yr BP) [8]. Most records document a warming trend in the early and mid-Holocene, reflecting the Holocene Thermal Maximum [9], followed by the onset of cooler, more stable late Holocene Neoglacial conditions between ca. 4,000 and 3,000 yr BP. These cool climate conditions generally persisted until the onset of recent warming linked to anthropogenic influences [7, 10–12]. Modes of climate variability, however, have been noted in the Subarctic region during this relatively stable climate interval. For example, examination of tree-ring records spanning the past ~400 years suggests that short-lived, sub-centennial, variations may be a key feature of climate in the Yellowknife, Northwest Territories area [5]. The hypothesized mechanism behind this variability is atmospheric teleconnections linked to the Pacific Decadal Oscillation (PDO; ca. 50–70 year periodicity), a phenomena whereby waters of the northeastern Pacific Ocean periodically shift between a positive PDO (warm) phase and a negative PDO (cool) phase [13]. This variability has been linked to periodic changes in total solar irradiance (TSI), the amount of solar radiative energy incident on the Earth’s atmosphere, that results in the observed PDO phenomena through ocean-atmosphere amplification processes [14]. Evidence of PDO influence has been observed in continental and oceanic climate records from the west coast of North America (e.g. [15, 16]). Along with the Atlantic Multidecadal Oscillation, the PDO may strongly influence multidecadal drought patterns throughout the continent [17]. Despite continent-wide recognition of these oceanic influences, studies focused on their inland extent into the climatically sensitive Subarctic region are limited. In this region, there is a need to develop methods to extend high-resolution climatic analyses to longer intervals than is possible with tree ring records (e.g. beyond ~400 years) to determine whether centennial and sub-centennial-scaled climate variability may be a more permanent feature of the Canadian Subarctic.

Here, we present a high-resolution analysis of climate proxy records archived in a well-dated (via radiocarbon) lacustrine sediment core spanning the past ca. 3300 yr BP from the treeline region in central Northwest Territories, Canada. We use proxies for lake ecology (diatoms) and catchment processes (sediment grain size data) to evaluate climatic trends and periodicities affecting the study area. Danny’s Lake was a suitable location to carry out the research because core records from this lake archive a sub-decadal-scale paleoenvironmental record, and previous work has shown the lake to be a sensitive site for tracking Holocene vegetation and catchment dynamics [7, 18]. The period from ca. 3300 yr BP to the present-day was chosen for this study because the late Holocene Neoglaciation of northwestern North America had become established by then [7, 19, 20], offering a stable time interval for study. High-resolution diatom and sediment grain size data were analysed using time series analysis techniques (spectral, wavelet) and the results of these analyses were compared to a previously published TSI record [21] to explore potential links between Pacific Ocean climate variability and climate in the Canadian Subarctic.

## Study site

**Fig 1:**
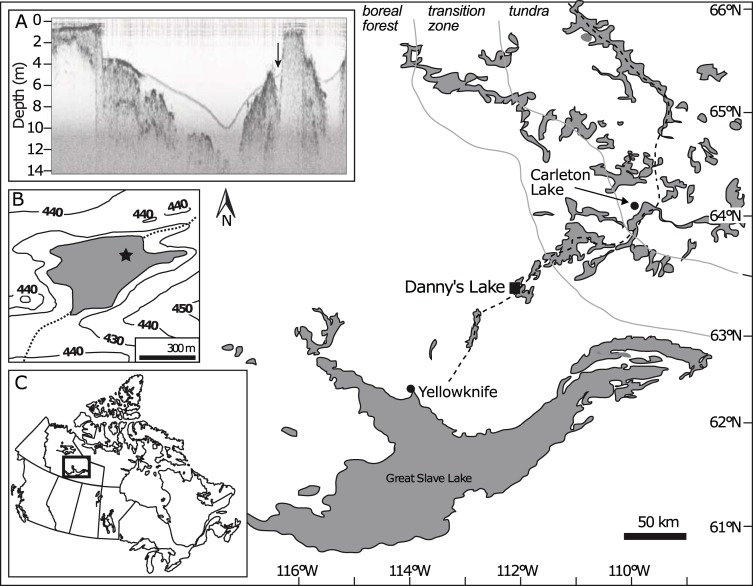
Map showing the location of Danny’s Lake in the central Northwest Territories, Canada. The Tibbitt to Contwoyto Winter Road (dotted line) and the relative position of boreal to tundra ecotone (grey lines) are shown. Inset figures show: (A) bathymetric transect of Danny’s Lake showing coring location with arrow. (B) Topographic map of Danny’s Lake with elevation contours in metres above sea level. Coring site shown with star. (C) The study area within Canada.

Danny’s Lake is located 40 km southwest of the modern treeline in Subarctic Canada (63.48°N, 112.54°W; Fig 1). It is a small polymictic lake with a surface area of 0.19 km^2^ and a maximum depth of 10 m (Fig 1A and 1B) that is well-mixed year round [22], lying in a region of poorly developed cryosols. No streams or rivers contribute water to Danny’s Lake, however an ephemeral inlet is located at the northeastern-most part of the lake, with an outlet at the southwest corner (Fig 1B). Water property data collected during summer 2011 indicates that there is no summer thermal stratification (Table 1).

**Table 1:** Water property data measured at Danny’s Lake, Northwest Territories, in winter 2010 and summer 2011. A Kemmerer water sampler was used to collect lake-bottom water. Measurements were taken using a hand-held YSI multiprobe.

	Dissolved oxygen mg/L		Conductivity (μS)		Water temperature (°C)		pH	
	Winter 2010	Summer 2011	Winter 2010	Summer 2011	Winter 2010	Summer 2011	Winter 2010	Summer 2011
Surface	9.91	8.74	12.8	34.8	0.2	14.5	6.61	8.1
Bottom	1.23	6.5	32.8	46.1	3.3	14.4	-	8.2

The area surrounding Danny’s Lake is characterized by a gently sloping topography underlain by discontinuous permafrost. Vegetation in this region is characterized by boreal forest transitioning northward into tundra, with the treeline boundary approximating the summer position of the Arctic Front [23]. In the study region, the boreal forest is dominated by black spruce (*Picea mariana*) and white spruce (*P*. *glauca*), with a tamarack (*Larix laricina*) and pine (*Pinus L*.) component. There are no precipitation data for the Danny’s Lake area. However, Yellowknife, 200 km to the southwest, has an average annual precipitation of 289 mm [24], and alternates between long, cold winters (January average minimum temperature -31.3°C) and brief, cool summers (July average maximum temperature 20.7°C). Precipitation peaks in August with an average 41 mm of rain [24].

## Methods

### Subsampling and chronology

A 116.2-cm long sediment core was retrieved from a 4.4 m deep sub-basin within Danny’s Lake using a custom-designed two-faced freeze corer in March 2010 [22]. The two sediment faces were transported frozen to Carleton University for analysis. Sediment faces were sectioned into 1.0 mm-thick subsamples using a custom-built sledge microtome [25]. To correlate the two sediment faces, sedimentological successions in each face were subjected to loss on ignition (LOI) and magnetic susceptibility (MS) analyses [22]. Sedimentological analyses (end-member mixing analysis) of the Danny’s Lake sediment core suggested no homogenization or re-working in the sediment core at 2-mm resolution [18], therefore we interpret bioturbation to be minimal or absent. Sediment re-deposition caused by wave activity is unlikely to have affected the sediment core owing to the water depth (4.4 m) and a coring location far from the shoreline.

**Fig 2:**
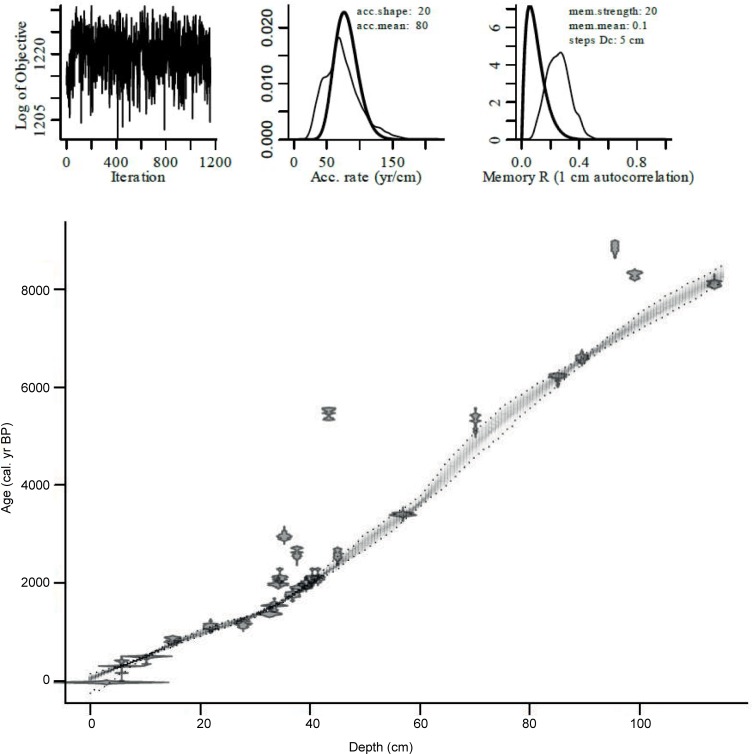
Bayesian age-depth model for Danny’s Lake constructed using Bayesian software Bacon [32, 33]. The main plot shows the age distributions of calibrated ^14^C dates and the grey-scale age-depth model indicates precisely dated sections of the chronology in darker grey, while lighter grey areas indicate less precise sections. The upper panel shows the stability of the Markov Chain Monte Carlo runs (1200 iterations); the prior (thick line) and posterior (thin line) distribution for the accumulation rate (yr/cm), and; the prior (thick line) and posterior (thin line) for the dependence of accumulation rate between sections.

The entire Danny’s Lake sediment core spans ca. 8000 yr BP to present-day and has a robust age model (Fig 2) that has been described previously [7, 18, 26]. This age model was achieved by sampling bulk sediment from 25 intervals spanning the entire sediment core that were then dated by radiocarbon accelerator mass spectrometry (AMS; Table 2). All samples underwent a standard hydrochloric acid (HCl) wash to remove carbonate material. A date of 430 years was obtained for the top of the sediment core therefore a freshwater reservoir effect (FRE) of 430 years was subtracted from all radiocarbon dates prior to calibration (cf. [27, 28]). Our uniform application of the surface FRE through the entire sediment core is justified following recent work in the study area that suggested a close match between surface FRE and at-depth FRE using tephra as an independent chronometer [29]. We attribute the 430-year FRE offset to represent in-wash of organic material from adjacent lands harboring old carbon [26]. Radiocarbon ages were calibrated using Calib version 6.1.0 [30] and the IntCal09 calibration curve [31]. The age-depth model for the Danny’s Lake sediment core was generated using the Bayesian software Bacon (Fig 2; Table 2) [32, 33]. The age model places the most recent ca. 3330 cal yr BP (e.g. the interval when the modern climate regime was established) in the upper 56.3 cm of the Danny’s Lake sediment core and these sediments are the focus of this study. Each 1-mm sample was therefore modelled to correspond to ca. 6 years of sediment accumulation (e.g. 3300 cal yr BP / 56.3 cm).

**Table 2:** Radiocarbon results for Danny’s Lake including the sample depth, fraction of modern carbon (F^14^C), radiocarbon ages uncorrected for the FRE (ΔR), radiocarbon ages corrected for the FRE, and calibrated dates based on the corrected age. The FRE corrected dates were calibrated using Calib version 6.1.0 [30] and the IntCal09 calibration curve [31] and calibrated ranges represent a relative area of greater than 95% (some values are composite). Outliers are shown in bold. Samples were dated at the 14CHRONO Centre for Climate, The Environment, and Chronology at Queen’s University, Belfast, United Kingdom.

				^14^C age BP (1σ)				
Lab ID (UBA-)	Depth (cm)	F^14^C		Uncorrected		Corrected ΔR = 430		Corr. cal yr BP (2σ)
17359	5.7	0.9174	± 0.0024	693	± 21	263	± 21	284–424
17360	10.2	0.8991	± 0.0026	855	± 23	425	± 23	462–519
16543	15.0	0.8475	± 0.0024	1329	± 23	899	± 23	740–908
17361	21.9	0.8177	± 0.0025	1617	± 25	1187	± 25	1055–1177
17431	27.8	0.8134	± 0.0021	1659	± 21	1229	± 21	1072–1257
16544	32.6	0.7878	± 0.0025	1916	± 25	1486	± 25	1315–1408
20377	33.5	0.7728	± 0.0023	2071	± 24	1641	± 24	1419–1611
**20378**	34.2	0.7643	± 0.0023	2159	± 24	1729	± 24	1566–1703
**17929**	34.5	0.7551	± 0.0026	2257	± 26	1827	± 26	1700–1825
**20376**	35.3	0.7726	± 0.0027	2073	± 28	1643	± 28	1417–1614
20375	36.8	0.7559	± 0.0024	2248	± 25	1818	± 25	1697–1822
**17432**	37.6	0.7182	± 0.0029	2659	± 32	2229	± 32	2152–2335
20374	38.4	0.7424	± 0.0023	2392	± 25	1962	± 25	1865–1953
20373	39.3	0.7373	± 0.0030	2448	± 33	2018	± 33	1885–2059
17930	40.4	0.7281	± 0.0024	2549	± 26	2119	± 26	2002–2152
20371	41.4	0.7276	± 0.0025	2554	± 28	2124	± 28	2002–2154
**20372**	43.3	0.5459	± 0.0020	4863	± 29	4433	± 29	4877–5276
16545	45.0	0.6960	± 0.0021	2912	± 24	2482	± 24	2459–2717
16546	56.9	0.6385	± 0.0020	3604	± 25	3174	± 25	3361–3446
16547	70.1	0.5340	± 0.0034	5039	± 51	4609	± 51	5057–5471
16548	85.1	0.4837	± 0.0017	5834	± 29	5404	± 29	6180–6286
17931	89.5	0.4604	± 0.0019	6231	± 34	5801	± 34	6496–6674
**16439**	95.5	0.3643	± 0.0014	8112	± 32	7682	± 32	8412–8541
**17932**	99.1	0.3871	± 0.0018	7623	± 38	7193	± 38	7940–8111
16440	113.6	0.3956	± 0.0015	7450	± 30	7020	± 30	7792–7935

### Diatom analyses

On average, one sample every 5 mm in the Danny’s Lake sediment core (e.g. 30-year sample resolution) was allocated to diatom analyses. Occasionally, sample spacing varied due to sample availability. A modified version of the method reported by Battarbee [34] was used to process diatom samples. Sediments were treated with 10 ml of 35% H_2_O_2_ and then heated in an 80°C water bath for six hours to digest organic material. One ml of 10% HCl was then added to dissolve carbonates. The resulting diatom slurries were rinsed with deionized water and a few drops of ammonia were added to the final wash to keep any remaining clay particles in suspension. Slurries were then pipetted onto coverslips, allowed to dry, and adhered to slides using Naphrax®. Identification and enumeration was carried out using an Olympus BX51 binocular light microscope at 1000X magnification under oil immersion. Taxonomy followed Krammer and Lange-Bertalot [35], updated to reflect present-day naming conventions [36] (see Table 3). On average, 449 diatom valves (maximum 540; minimum 392) were identified on each slide to obtain statistically significant counts [37]. It is possible that the occasional enumeration of chain-formed taxa (e.g. *Aulacoseira*) introduced variability into the dataset. The authors assume that this variability is statistically minimal due to the high number of specimens quantified in each sample. Chrysophyte cysts were also enumerated in each sample.

**Table 3:** Diatom species shown in the stratigraphic diagram of the Danny’s Lake sediment core. Taxonomy was updated to reflect present-day naming conventions [36].

Species name in the present publication (updated)	Original taxonomy after Krammer and Lange-Bertalot [35]	Authority	Species group in Fig 4
*Aulacoseira alpigena*	*Aulacoseira alpigena*	(Grunow) Krammer	n/a
*Aulacoseira distans*	*Aulacoseira distans*	(Ehrenberg) Simonsen	*Aulacoseira* complex
*Aulacoseira lacustris*	*Aulacoseira lacustris*	(Grunow) Krammer	*Aulacoseira* complex
*Aulacoseira perglabra*	*Aulacoseira perglabra*	(Østrup) Haworth	*Aulacoseira* complex
*Discostella pseudostelligera*	*Cyclotella pseudostelligera*	Hustedt	*Discostella stelligera* complex
*Discostella stelligera*	*Cyclotella stelligera*	Cleve & Grunow	*Discostella stelligera* complex
*Cyclotella ocellata*	*Cyclotella ocellata*	Pantocsek	n/a
*Aulacoseira subarctica*	*Aulacoseira subarctica*	(O Müller) Haworth	n/a
*Cyclotella tripartita*	*Cyclotella tripartita*	Håkansson	n/a
*Achnanthidium minutissimum*	*Achnanthes minutissima*	Kützing	n/a
*Staurosira construens* var. *venter*	*Fragilaria construens* f *venter*	(Ehrenberg) Hustedt	*Pseudostaurosira* complex
*Staurosirella pinnata*	*Fragilaria pinnata*	Ehrenberg	*Pseudostaurosira* complex
*Pseudostaurosira pseudoconstruens*	*Fragilaria pseudoconstruens*	Marciniak	*Pseudostaurosira* complex
*Pseudostaurosira brevistriata*	*Fragilaria brevistriata*	(Grunow) Williams and Round	n/a
*Staurosira construens* var. *exigua*	*Fragilaria construens* var. *exigua*	(Smith) Kobayasi	n/a
*Achnanthes levanderi*	*Achnanthes levanderi*	Hustedt	*Psammothidium* complex
*Achnanthes pusilla*	*Achnanthes pusilla*	(Grunow) De Toni	*Psammothidium* complex
*Brachysira brebissonii*	*Anomoeoneis brachysira*	Ross	n/a
*Karayevia suchlandtii*	*Achnanthes suchlandtii*	(Hustedt) Bukhtiyarova	n/a
*Brachysira microcephala*	*Anomoeoneis vitrea*	(Grunow) Ross	n/a
*Eunotia faba*	*Eunotia faba*	(Ehrenberg) Grunow	n/a
*Staurosira construens var*. *binodis*	*Staurosira construens var*. *binodis*	(Ehrenberg) Hamilton	n/a
*Nitzschia fonticola*	*Nitzschia fonticola*	(Grunow) Grunow	n/a
*Tabellaria flocculosa*	*Tabellaria flocculosa*	(Roth) Kützing	n/a

Diatom data were plotted on a stratigraphic diagram that was created using the C2 program [38]. Only diatom species with relative abundances of at least 2% in one or more samples were included in the stratigraphic diagram. Taxa were grouped based on similar trends and ecology among species [4] (Table 3). Shannon Diversity Index (SDI) [39] and the ratio of chrysophyte cysts to diatoms (C:D) were calculated to reconstruct the trophic status of the lake over time (e.g. a high C:D ratio may indicate nutrient-poor waters [40]). Stratigraphically constrained incremental sum of squares cluster analysis (CONISS) was carried out using the ‘rioja’ package in R [41–43]. The number of zones was determined by comparison to a broken stick model [44].

### Sedimentology

Particle size was determined every 2 mm (e.g. 12-year sample resolution). Following Murray [45] and van Hengstum *et al*. [46], 10% H_2_O_2_ was added to sub-samples in an 80°C water bath to remove organic matter prior to analysis. Carbonates generally represented <1% of the dried sediment mass in the Danny’s Lake core [47], thus we consider their impact on grain size to be minimal. The sediments were then analysed using a Beckman Coulter LS 13 320 Laser Diffraction-Particle Size Analyzer with a Universal Liquid Module. Organic, carbonate and minerogenic contents of the samples were determined every 10 mm of the sediment core by LOI analysis using a Thermo Scientific Thermolyne Benchtop Muffle Furnace (Model: F48025-60-80) at temperatures of 550 **°**C and 950 **°**C for four hours and two hours, respectively [48]. The only exception was the top 10 cm of the core, where sediment was not of sufficient quantity to carry out LOI analysis. Magnetic susceptibility was measured along the intact freeze core using a Bartington MS2B sensor set to low frequency [49]. Results are presented in standard international units. Sedimentological interpretations for Danny’s Lake are based on recent work that examined the relationship between lake sediments, watershed processes and hydrology in this subarctic region [18].

### Time series analyses

Time series analyses were conducted on selected diatom data (ca. 30-year resolution) and the coarse silt fraction (sedimentology data; ca. 12-year resolution) from the Danny’s Lake sediment core. Diatom groups (discussed in further detail below) were chosen based on their sensitivity to physio-chemical parameters, their span of both benthic and planktic habitats, along with their dominance in the paleorecord (together comprising 23–53% of the diatom assemblage). Particle size shifts provide information about watershed processes; changes have been linked to changing local snow pack and spring freshet conditions and explain a large percentage of the variance in sediment record in the Danny’s Lake sediment core [18]. Two different time series analyses techniques were conducted; spectral analysis, which identifies statistically significant periodicities in the data, and wavelet analysis, which shows the timing and duration of periodicities through the examined interval. Previously published TSI data [21] were also included. Diatom data and sedimentological data were detrended and interpolated (to exactly 30 years and 12 years, respectively) to ensure equal sample spacing for time series analysis. In addition, diatom raw data counts were corrected for statistical biasing and closure issues [50] by adding an arbitrary constant followed by the calculation of the geometric mean for each sample. The relative abundance was then divided by the geometric mean of that sample, and finally each species was expressed in the log (base 10) form. The TSI record [21] was analysed at its original 5-year sampling resolution. Spectral analysis was carried out using the software Past (v3.12) [51], which is based on the Lomb-Scargle Fourier Transform. A parametric approach was used to assess the significance of spectral peaks (90%, 95%, and 99% χ^2^ false-alarm levels) against a realistic null hypothesis of red (auto-correlated) noise [52]. Wavelet analyses were conducted using R package ‘biwavelet’ [53].

One of the most important influences on lake hydrology and algal ecology in the Subarctic region is changes to the timing and duration of seasonal lake ice cover [12, 54], which can impact the timing and availability of diatom habitats, lake stratification and other water properties [55]. Three species of diatoms were selected for time series analysis. The first is planktic, *Aulacoseira alpigena* that comprise $\bar{x}$ = 26.7% (SD = 8.3) of the diatom assemblage. The genus *Aulacoseira* has heavily-silicified frustules and requires strong convective lake mixing to remain within the water column [56, 57]. It is generally adapted to lower light conditions and decreased pH levels that may be associated with greater runoff from the surrounding terrain [58]. The second species is benthic, higher pH-preferring *Pseudostaurosira brevistriata*, a fast-growing opportunistic species with the capacity to thrive during short growing seasons under harsh climatic conditions [59], which comprise $\bar{x}$ = 6.1% (SD = 2.0) of the diatom assemblage. This species has been used as an indicator of seasonal ice cover [59, 60]. This species is tolerant of water columns with higher light conditions [61], and has been reported to have a generally higher pH optimum tolerance than *A*. *alpigena* [62, 63]. Thirdly, benthic, periphytic generalist *Achnanthidium minutissimum* [59, 64], which comprise $\bar{x}$ = 5.2% (SD = 2.7) of the diatom assemblage and exhibit some tolerance to lower pH conditions [65]. This species is characteristic of disturbed conditions and is able to rapidly invade vacant niches created by hydrologic changes in physico-chemical parameters [66].

## Results

### Sedimentology

**Fig 3:**
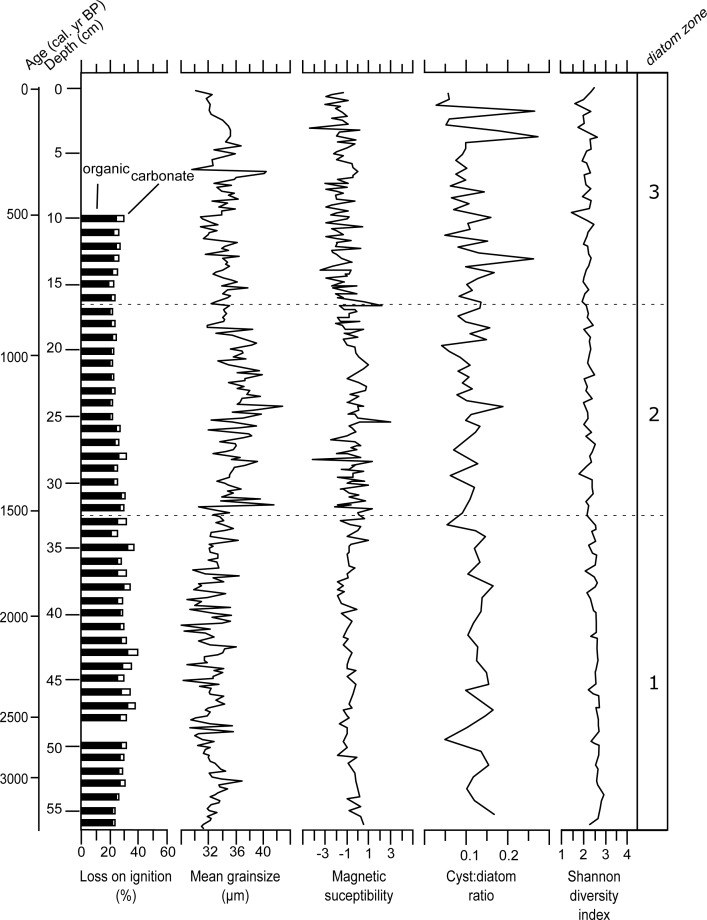
Sedimentological and ecological parameters for the Danny’s Lake sediment core.

On average, the examined interval (56.3 to 0.5 cm) of the Danny’s Lake sediment core was composed of 25.7% (SD = 3.6) organic content, 3.3% (SD = 1.5) carbonates (which may represent interstitial water loss from clays in the sediment matrix) and the remaining 71% (SD = 4.7) was minerogenic in origin (Fig 3; S1 Table). The average sediment size fraction (S2 Table) was coarse and very coarse silt, ranging from 32 μm (SD = 1.8) throughout the lowermost examined interval (56.3 to 33.3 cm), increasing to $\bar{x}$ = 35.8 μm (SD = 2.4) in the middle (32.6 to 17.5 cm) and then decreasing to $\bar{x}$ = 34.1 μm (SD = 1.93) in the uppermost sediments (17 to 0.5 cm). Low frequency MS values were consistently below 0 in the lowermost interval $\bar{x}$ = -0.76 (SD = 0.62), becoming increasingly variable in the middle ($\bar{x}$ = -0.4; SD = 1.1; n = 77), and then decreasing to $\bar{x}$ = -1.5 (n = 86; SD = 1.0) in the uppermost sediments (Fig 3).

### Diatoms

Diatoms were well preserved throughout the examined interval of the Danny’s Lake sediment core. Three major diatom assemblage zones were identified using stratigraphically constrained cluster analysis: D-1 (56.3 to 33.3 cm; ca. 3330 to 1540 cal yr BP), D-2 (32.6 to 17.5 cm; ca. 1500 to 860 cal yr BP) and D-3 (17 to 0.5 cm; ca. 840 cal yr BP to present-day). There were few pronounced changes in the diatom assemblage over time as shown by the SDI data, which ranged from 1.7 to 2.7 throughout the examined interval (Fig 3). The C:D ratio was stable for most of the lower sediments, but displayed notable changes in the upper sediments.

**Fig 4:**
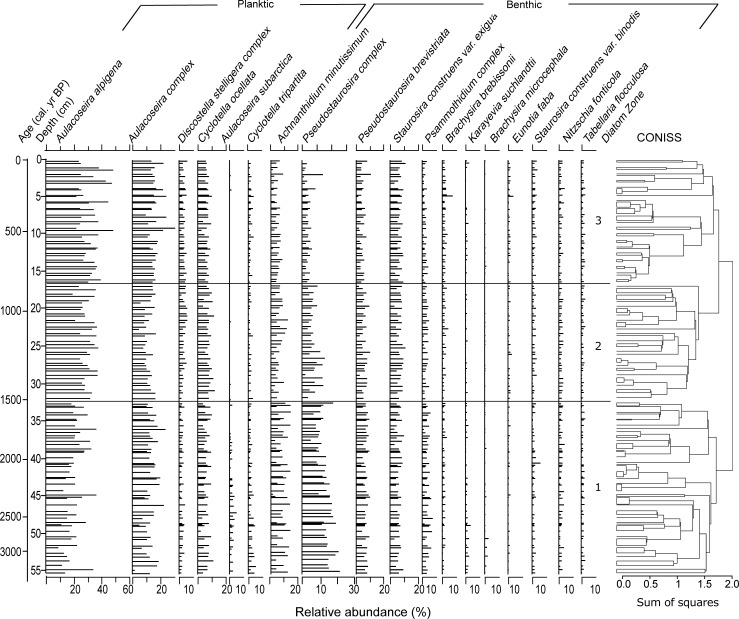
Diatom data from the Danny’s Lake sediment core, along with results of stratigraphically constrained cluster analysis (CONISS). Only diatom taxa that reached >2% relative abundance in at least one sample were plotted.

Overall, the diatom assemblage (Fig 4) was dominated by planktic *A*. *alpigena*, which had an average relative abundance of 20.5% (SD = 6.6) at the lowermost examined interval (D-1), increasing to at $\bar{x}$ = 29.7% (SD = 4.9) in interval D-2, and finally $\bar{x}$ = 32.1% (SD = 7.4) in the uppermost interval (D-3). Planktic *Aulacoseira* complex along with *Cyclotella ocellata* occupied $\bar{x}$ = 13.1% (SD = 4.2) and $\bar{x}$ = 6.9% (SD = 1.9) of the overall assemblage, showing little variability between sampling intervals. Benthic diatoms included *Pseudostaurosira* complex and *A*. *minutissimum*, which had relative abundances of $\bar{x}$ = 12.1% (SD = 3.6) and $\bar{x}$ = 6.7% (SD = 3.0) in the lowermost examined sediments (D-1), decreasing to $\bar{x}$ = 3.3% (SD = 1.9) and $\bar{x}$ = 3.7% (SD = 1.5), respectively. The remainder of the diatom assemblage was comprised of species showing little change throughout the examined sequence; *Staurosira construens* var. *exigua* averaged $\bar{x}$ = 6.7% (SD = 1.7), *P*. *brevistriata* at $\bar{x}$ = 6.1% (SD = 2.0), *Psammothidium* complex at $\bar{x}$ = 2.9% (SD = 1.4) and *Discostella stelligera* at $\bar{x}$ = 2.9% (SD = 1.2). Diatom taxa occupying less than 2% of the total assemblage are not discussed here, however all data are included in S3 Table.

### Time series analyses of diatom and sedimentology data

**Fig 5:**
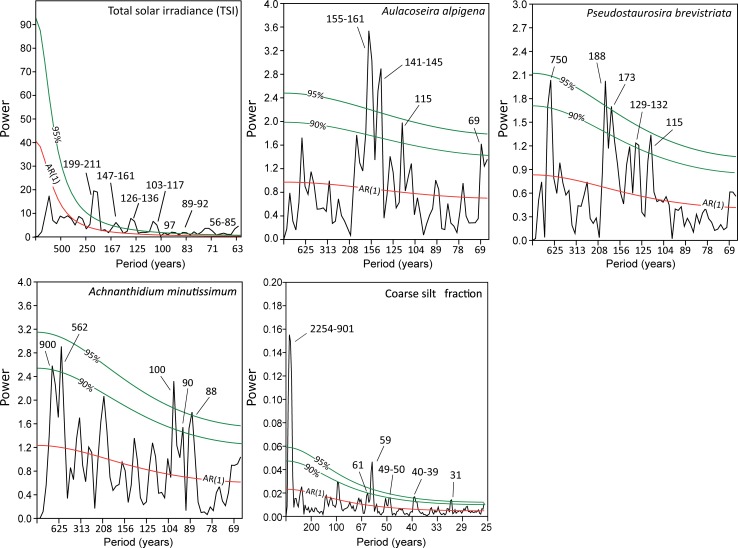
Spectral results for total solar irradiance (TSI), three key diatom species and the coarse silt fraction in the Danny’s Lake sediment core. Red and green lines indicate confidence intervals. Spectral peaks are noted.

Spectral analysis identified various periodicities in the TSI record, spanning the ca. 56-year range to the ca. 211-year range (Fig 5). The diatom data showed statistically robust (>95% confidence interval) variations in the ca. 141–145 and ca. 155–161-year range in *A*. *alpigena*, the ca. 173 and 188-year range for *P*. *brevistriata* and the ca. 88 and 100-year ranges for *A*. *minutissimum* (Fig 5). Variability in the range of ca. 900, 750, 562, 129–132, 115, 90 and 69 years was also noted at the >90% interval in the diatom data. The coarse silt sediment fraction (examined at a higher resolution than the diatom data) revealed statistically robust (>95% confidence interval) variations in the ca. 31, 39–40, 59 and 901–2254-year ranges. Variability in the range of ca. 61 and ca. 49–50 years was also noted at the >90% confidence interval in the sedimentology data.

**Fig 6:**
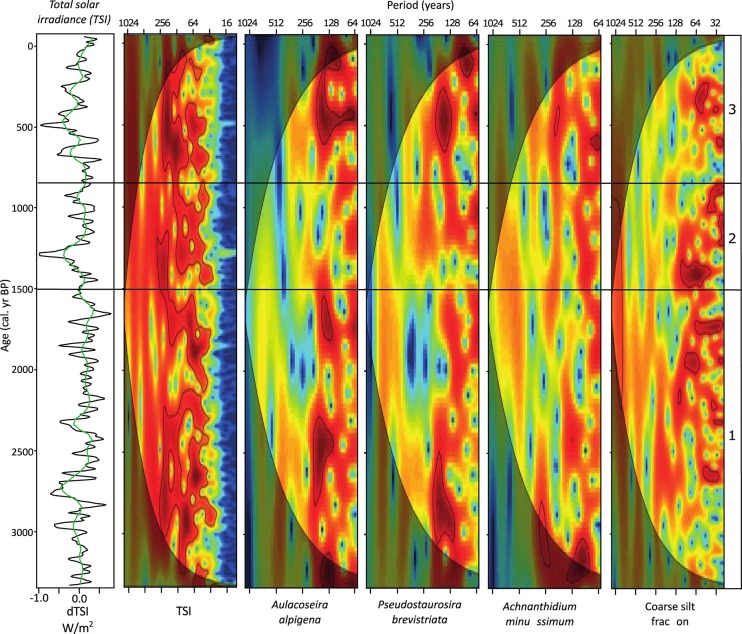
Wavelet results for total solar irradiance (TSI), three key diatom species and the coarse silt fraction of the Danny’s Lake sediment core. High values (red color) are assigned to areas where the indicated periodicity is persistent. Low values (blue color) indicate lack of periodicities at the given wavelength and time period, and faded region is outside of the cone of influence. The black line surrounding the high values (red color) indicates 95% significance against red noise background.

Wavelet analyses (Fig 6) showed that periodicities identified in the TSI record (Fig 5) were present intermittently throughout the examined interval. One of the most prominent periodicities in the TSI record was in the range of ca. 150–200 years, which was present from ca. 3000–2200 cal yr BP, and intermittently between ca. 1400 and 200 cal yr BP. An additional TSI periodicity in the range of ca. 60 years was present intermittently throughout the majority of the examined interval. Wavelet analyses on diatom data (Fig 6) showed that identified spectral peaks (Fig 5) occurred occasionally through the study interval. There were statistically significant periodicities in the range of ca. 140–188 years between ca. 3000 and 2300 cal yr BP in *A*. *alpigena* and *P*. *brevistriata*. Periodicities in this range re-occurred from ca. 1800–1500 cal yr BP in *A*. *alpigena*. There was also a brief interval between ca. 3200 and 3000 cal yr BP when *A*. *minutissimum* had significant periodicities in the ca. 88 and ca. 100-year range. No statistically significant periodicities occurred in the examined diatom data in the D-2 interval. However diatom groups showed significant periodicities during D-3, notably, periodicities in the range of ca. 140–188 years between ca. 600 and 200 cal yr BP in both *A*. *alpigena* and *P*. *brevistriata*. At ca. 450 cal yr BP, the periodicities in *A*. *alpigena* appeared to be ‘stacked’, and spanned the range of ca. 200–64 years. The coarse silt sedimentology data for the Danny’s Lake sediment core showed only periodicities in the <100-year range, in particular a ca. 60-year periodicity, which occurred discontinuously though the interval of ca. 1900–1300 cal yr BP, and again from ca. 400–250 cal yr BP.

## Discussion

Overall, the diatom assemblage contained in the Danny’s Lake core (Fig 4) was similar to the records in other nearby boreal/treeline lakes that are ice-covered for several months of the year (e.g. Slipper Lake, located 50 km north of the present-day treeline; [67]). The most pronounced long-term change in the record was the gradual increase in planktic (*Aulacoseira*) diatoms, and the subsequent decrease in benthic (*Pseudostaurosira*) diatoms in the uppermost sediments. In interval D-1 (ca. 3330 to 1540 cal yr BP), benthic diatom group *Pseudostaurosira* are at the highest abundance. This benthic species group can better tolerate conditions of increased ice-cover, characterized by lower light penetration and cooler water temperatures [68], therefore it is possible that interval D-1 was a slightly cooler than conditions later in the record. These cooler climate conditions may correspond to the onset of the late Holocene mid-Neoglacial Tiedemann advance observed in the North American Cordillera [3, 67, 69, 70].

A slight shift in the overall diatom assemblage was noted in interval D-2 (ca. 1500 to 860 cal yr BP). Notably, the declining abundances of periphytic benthic taxa such as *A*. *minutissimum* and *Pseudostaurosira* complex, and increasing abundances of planktic *A*. *alpigena*, and *Aulacoseira* complex species (Fig 4), suggested that the lake became more favourable for planktic relative to benthic species. Possible causes for this shift include lower light penetration in the lake and/or a deepening of water depth. These conditions could have been brought on by greater regional precipitation that led to increased erosion into the watershed. Interestingly, this time interval corresponds to intensified northwestern North American climate cooling as evidenced by a cooling in nearby chironomid-based temperature reconstruction [6] and glacial advances at higher elevations [71]. Increased *Pinus banksiana* in the Danny’s Lake sediment core during that time [7] could also be the result of this regional climate cooling. Increases in *P*. *banksiana* are sometimes associated with a vegetation succession related to recovery from fire activity [72]; however, no increase in charcoal was noted in the Danny’s Lake sediment core through this interval [7], which suggests that the increase in *P*. *banksiana* was more likely related to regional cooling. An overall increase in the sediment size fraction through this interval, along with highly variable MS values (Fig 3) suggests that there was increased erosional activity in the watershed and more runoff into the lake [18], which corresponds to a sharp increase in accumulation rate in several nearby lake records [26], and is consistent with interpretations of an increase in regional annual precipitation levels (derived from pollen records) during this period [73]. The continued decline of alkaliphilic *Pseudostaurosira* species during this interval may be related to a gradual lowering of lake pH over time as the result of extended winter ice cover seasons, also related to regional cooling [74]. *D*. *stelligera* complex declines slightly in abundance at ca. 1000 cal yr BP, which may be a response to warming associated with the Medieval Climate Anomaly (MCA), which has been inferred in other studies from the region [4] as well as throughout Arctic Canada between ca. 1100 to ca. 900 yr BP [73].

We interpret the most recent interval, D-3 (ca. 840 cal yr BP to present-day; Fig 4) as a return to more stable hydroecological conditions. The continued decrease of *Pseudostaurosira* complex, concurrent with an increase of planktic *A*. *alpigena* (Fig 4) suggests a decrease in year-to-year ice cover and development of favorable hydroecologic lake overturn conditions. Decreased sediment size fractions suggest less erosion in the watershed. In contrast, MS values remain highly variable, indicative of ongoing perturbations in the watershed (Fig 3). It is possible that lake productivity may have undergone fluctuations and/or increases during this interval as suggested by the fluctuations in the C:D ratio. Evidence of fluctuating lake productivity is also supported by previous research on the Danny’s Lake sediment core related to an increase in aquatic algae *Pediastrum* and a decrease in the C:N ratio during that interval [7, 47]. At our 30-year diatom sample resolution, we noted no increases in the relative abundance of *Aulacoseira* spp. between ca. 750 to ca. 200 cal yr BP that would have suggested a cooling influence of the Little Ice Age (e.g. [4, 67]). Similarly, in the uppermost seven sediment samples that are modelled to have been deposited from the 19^th^ century to today, we noted no diatom shifts that could be attributed to 19^th^ century warming or recovery from the Little Ice Age. These results are a contrast to an analysis of sediments of the same age from nearby Slipper Lake (located 50 km north of the present-day treeline), where diatom flora of underwent a dramatic shift from benthic-dominated to planktic-dominated assemblages at ca. 100 cal yr BP [67]. The results of other studies in this region of the Canadian Arctic, as well as from Finland, also provide evidence of a similar shift following the Little Ice Age, often associated with an increase in the relative abundance of *D*. *stelligera* [4, 12, 56, 67]. The failure of *D*. *stelligera* to record these widely recognized 19^th^ century changes at Danny’s Lake may be related to the ecological complexity of the *Discostella* taxa. Recent work has shown that the response of *D*. *stelligera* to recent warming is not universal, and that the ecology of *Discostella* taxa are dependent on various physical, nutrient and chemical parameters in lakes [75].

### Time series analyses

High-resolution sampling of the diatom (30-year resolution) and coarse silt sedimentology data (12-year resolution) paleo-records in the Danny’s Lake sediment core allowed for unique insights into long-term climate variability in the Canadian Subarctic. Taking into consideration the ecology of the diatom species that were selected for time series analyses (Section 3.4), the periodicities in diatom assemblage suggest repeated variations in lake hydroecology and physiochemical parameters (e.g. productivity, lake convection, pH, light penetration) that persisted for sometimes extended intervals during the last 3300 years of the late Holocene (Figs 5 and 6). It is likely that many documented changes in diatom abundance were related to changes in the timing and duration of seasonal ice cover; one of the most dominant controls on lake hydrology, ecology and physiochemical parameters in this region [12, 54, 55]. As a whole, the periodicities observed in proxy data from the Danny’s Lake sediment core share similarities with the TSI record (Fig 5), notably in the range of ca. 140–160, 100, 90 and 60 years.

Signal periodicities in the range of ca. 188-years are an intermittent feature in the *P*. *brevistriata* record between ca. 3000 and 2300 cal yr BP in (Figs 5 and 6). Based on the ecology of this diatom species, this variability may be linked to changes in seasonal ice cover, pH and possibly lake convection. The observed ca. 188-year variability could be related to the ~200 year Suess Cycle, which is also identified as a significant periodicity within the TSI record (199–211 years; Fig 5). Similar variability has previously been linked to a westward shift/weakening of the Aleutian Low, a low-pressure air mass over the Northern Pacific Ocean [15, 76, 77]. The recognition of Suess-type periodicities in the Danny’s Lake sediment core may, therefore, suggest a periodic eastward shift (and/or decreased intensity) of the North Pacific High pressure system, resulting in changes to atmospheric circulation (e.g., the jet stream) that occasionally allow for air masses impacted by this Pacific Ocean variability to penetrate far inland into the Canadian Subarctic.

Spectral and wavelet results in the Danny’s Lake diatom record (Figs 5 and 6) reveal statistically significant periodicity in *A*. *minutissimum* in the ca. 88 and 100-year range that was present at the beginning of the examined record, between ca. 3200 and 3000 cal. yr BP. If our hypotheses regarding solar influence in the Canadian Subarctic are correct, these periodicities may be indicative of the influence of the Gleissberg Cycle on lake hydroecology, in particular occasional shifts between available diatom habitats (e.g. benthic- and planktic-) and possibly changes to the duration/timing of seasonal ice cover. The well-documented Gleissberg sunspot cycle [78, 79] was initially assumed to have a cyclicity of 88 years [80–82] but is now known to be comprised of a high-frequency band of 50–80 years and low-frequency band of 90–140 years [83]. Thus, observed periodicities in the range of ca. 141–145 in *A*. *alpigena*, along with ca. 129–132 and 115-year periodicities noted in *P*. *brevistriata* (at >90% confidence interval; Fig 5) may correspond to the low-frequency band of the Gleissberg Cycle.

Within the higher-resolution coarse silt sedimentology data, one of the most pronounced periodicities in the Danny’s Lake record was in the ca. 59-year range (Fig 5), suggesting regular perturbations in watershed processes, local snow pack and spring freshet [18]. These sedimentological data may correspond to the pentadecadal frequency of the PDO [84, 85]. The paired occurrence of this PDO-range periodicity in the sediment and TSI records suggests a possible connection between lake-wide hydroecological response and this external driver. Given what is known about the influence of the PDO based on climate records from the west coast of North America (e.g. [15, 16]), as well as from tree ring data in the Yellowknife area 200 km to the south of our study site [5], it is possible that recognition of this periodicity in the Danny’s Lake sediment core is indicative of the propagation of Pacific Ocean variability into the Canadian subarctic through changes in air mass circulation patterns and their influence on the jet stream [15, 86]. Periodicities in the range of ca. 69 years in diatom species *A*. *alpigena* (at the 90% confidence interval) may suggest a potential ecological link to this PDO-type variability at Danny’s Lake. However this interpretation is tenuous because it is near to the statistical limitation associated with the Nyquist frequency of the data (60 years). Future work that prioritizes higher decadal-scale sampling resolution will provide additional insight on the influence of the PDO in the Canadian Subarctic.

Some periodicities that we identified in the spectral and wavelet data (Figs 5 and 6) are more difficult to interpret in the context of known paleoclimate drivers. For example, there are intermittent periodicities in the ca. 155–161-year range in *A*. *alpigena* and the 173-year periodicity in *P*. *brevistriata* (Fig 5) that are not temporally correlative to known PDO, Gleissberg or Suess cycles. Swindles *et al*. [87] interpreted similar periodicities observed in Holocene peatlands from Ireland as potentially being sub-harmonics of the ca. 23-year Hale frequency. Similar periodicities have also been reported from annually laminated sediment color records from the northeast Pacific Ocean [88]. In addition, variations in the range of ca. 900, ca. 750 and ca. 562 (present at the >90% confidence interval) are difficult to interpret; similar periodicities have been noted in Holocene vegetation changes in East Asia [89] and shifts in sediment transport and marine productivity in the Mediterranean [90].

The intermittent nature of many of the diatom periodicities over the past ca. 3300 yr BP in the Danny’s Lake sediment core is noteworthy, especially given the almost continuous presence of similar signals in the TSI record throughout this interval. For example, examined diatom species showed no significant periodicities during interval D-2 (ca. 1500–860 cal yr BP), despite the persistence of periodicities in TSI (Fig 6). This is in contrast to the detection of purported PDO- and Gleissberg-type variations in laminated marine sediments from the coast of the Pacific Ocean during that time [88]. Additional work is needed to determine whether the breakdown of diatom periodicities could be the result of shifts in regional air masses, increased precipitation or loss of lake sensitivity due to a physical, ecological or environmental factor (e.g. increase to lake level). Irrespective of the mechanism, this finding suggests that changes in lake hydroecology are not solely influenced by TSI.

Our diatom data show a potential link between climate in the Canadian Subarctic region and the Suess and Gleissberg cycles, whereas the coarse silt sedimentology data suggest a possible link to the PDO. At several times during the examined interval, several of these periodicities occur simultaneously. For example, at ca. 450 cal yr BP, the periodicities in *A*. *alpigena* appear to span the entirety of ca. 64–200-years (Fig 6). This interval coincides with the onset of the Little Ice Age, which is associated with the Maunder minimum of solar output. The strengthening of periodicities during this interval suggests that changes in solar output may have led to an amplified control of TSI on lake hydroecology during that time. A similar yet brief pattern is noted at ca. 1750 cal yr BP when *A*. *alpigena* displays periodicities in the range of ca. 140 years and the coarse silt fraction has a significant periodicity in the range of ca. 60 years (Fig 6). In this case, the paired occurrence of Gleissberg-type and PDO-type periodicities may provide some evidence to support the hypothesis that the Gleissberg Cycle may be the “pace-maker” of the PDO [91]. Unfortunately, it is not possible to use the time series analysis presented here to study periodicities leading into the present-day because any changes post A.D. 1800 are not detectable using wavelet results due to edge effect phenomena (see [92]) on the wavelet plot (Fig 6). Despite this limitation, it is reasonable to assume that the influence of large-scale ocean-atmosphere phenomena on the climate of continental Canada that occurred intermittently over the past ca. 3300 yr BP likely continued on a similar basis through this interval.

## Conclusions

The Danny’s Lake sediment core was an ideal dataset for studying long-term climate variability in the Canadian subarctic owing to the sensitivity of diatoms and coarse silt sedimentology data to short-lived hydroecological changes in the lake and the exceptionally high-resolution dating and sub-sampling of the sediment. Our results suggest that Pacific Ocean climate variability influenced climate in the Canadian Subarctic intermittently over the past ca. 3300 cal yr BP. We attribute intermittent changes in the relative abundance of key diatom species occupying a range of benthic and planktic niches to variations in ecological and physical parameters within the lake basin that were potentially driven by climatic influences associated with PDO, Gleissberg and Suess cycles. Our results provide an important contribution to understanding baseline climate conditions for the Canadian Subarctic and extend the known interval of influence of northeast Pacific climate variability to the Canadian Subarctic for much of the late Holocene. Continued work examining high-resolution lake sediment records from this region may permit a better understanding of the extent of Pacific influence on subarctic climate.

## Supporting information

S1 Table Loss-on-ignition data for the Danny's Lake sediment core. (XLSX)

S2 Table Sedimentological data for the Danny's Lake sediment core. (XLSX)

S3 Table Raw diatom counts for the Danny's Lake sediment core. This table uses the taxonomy of Krammer & Lange-Bertalot (1990).
(XLSX)
